# Immature morphological properties in subcellular-scale structures in the dentate gyrus of Schnurri-2 knockout mice: a model for schizophrenia and intellectual disability

**DOI:** 10.1186/s13041-017-0339-2

**Published:** 2017-12-12

**Authors:** Akito Nakao, Naoyuki Miyazaki, Koji Ohira, Hideo Hagihara, Tsuyoshi Takagi, Nobuteru Usuda, Shunsuke Ishii, Kazuyoshi Murata, Tsuyoshi Miyakawa

**Affiliations:** 1 Division of Systems Medical Science, Institute for Comprehensive Medical Science, Fujita Health University, 1-98 Dengakugakubo, Kutsukake-cho, Toyoake, Aichi 470-1192 Japan; 2 National Institute for Physiological Sciences, National Institutes of Natural Sciences, Okazaki, Japan; 3 Department of Food Science and Nutrition, Mukogawa Women’s University, Nishinomiya, Japan; 4 Institute for Developmental Research, Aichi Human Service Center, Kasugai, Japan; 5 RIKEN Tsukuba Institute, Tsukuba, Japan; 6 Department of Anatomy II, Fujita Health University School of Medicine, Toyoake, Japan

**Keywords:** Mouse model, Schizophrenia, Intellectual disability, 3D electron microscopy

## Abstract

Accumulating evidence suggests that subcellular-scale structures such as dendritic spine and mitochondria may be involved in the pathogenesis/pathophysiology of schizophrenia and intellectual disability. Previously, we proposed mice lacking Schnurri-2 (Shn2; also called major histocompatibility complex [MHC]-binding protein 2 [MBP-2], or human immunodeficiency virus type I enhancer binding protein 2 [HIVEP2]) as a schizophrenia and intellectual disability model with mild chronic inflammation. In the mutants’ brains, there are increases in C4b and C1q genes, which are considered to mediate synapse elimination during postnatal development. However, morphological properties of subcellular-scale structures such as dendritic spine in Shn2 knockout (KO) mice remain unknown. In this study, we conducted three-dimensional morphological analyses in subcellular-scale structures in dentate gyrus granule cells of Shn2 KO mice by serial block-face scanning electron microscopy. Shn2 KO mice showed immature dendritic spine morphology characterized by increases in spine length and decreases in spine diameter. There was a non-significant tendency toward decrease in spine density of Shn2 KO mice over wild-type mice, and spine volume was indistinguishable between genotypes. Shn2 KO mice exhibited a significant reduction in GluR1 expression and a nominally significant decrease in SV2 expression, while PSD95 expression had a non-significant tendency to decrease in Shn2 KO mice. There were significant decreases in dendrite diameter, nuclear volume, and the number of constricted mitochondria in the mutants. Additionally, neuronal density was elevated in Shn2 KO mice. These results suggest that Shn2 KO mice serve as a unique tool for investigating morphological abnormalities of subcellular-scale structures in schizophrenia, intellectual disability, and its related disorders.

**Electronic supplementary material:**

The online version of this article (10.1186/s13041-017-0339-2) contains supplementary material, which is available to authorized users.

## Introduction

Accumulating evidence suggests that abnormalities in subcellular-scale structures such as dendritic spine and mitochondria may be involved in the pathogenesis/pathophysiology of schizophrenia, bipolar disorder, autism spectrum disorder, and intellectual disability [1–4]. Spines are morphologically and biochemically discrete compartments that protrude from dendrites [5]. It has been reported that spine enlargement parallels long-term potentiation, whereas long-term depression is associated with spine shrinkage [6]. Notably, psychiatric disorders such as schizophrenia, autism spectrum disorder, and intellectual disability have been reported to be accompanied by disruptions in dendritic spine shape, volume, or number [1, 3]. Mitochondria play a central role in various cellular processes that include regulation of ATP production, intracellular Ca^2+^ concentration, and redox homoeostasis [7]. Through these functions, mitochondria control various neural processes [8]. It has been suggested that mitochondrial dysfunction underlies the pathophysiology of schizophrenia, bipolar disorder, and intellectual disability [2, 9].

Schnurri-2 (Shn2; also called major histocompatibility complex [MHC]-binding protein 2 [MBP-2], human immunodeficiency virus type I enhancer binding protein 2 [HIVEP2], or c-myc intron binding protein 1 [MIBP1]) was originally identified as a nuclear factor-κB (NF-κB) site-binding protein that binds tightly to the enhancers of MHC genes in the MHC regions of chromosome 6 [10]. Recent genome-wide association studies have identified a number of single-nucleotide polymorphisms in the MHC region associated with schizophrenia [11–15]. MHC class I proteins coded in this region are reported to play important roles in neural processes [16]. Genes in the MHC regions often harbor NF-κB-binding sequences in their promoter regions. Shn2 constitutively binds to NF-κB-binding sites to suppress NF-κB-dependent gene expression [17]. To induce an immune response, Shn2 detaches from the NF-κB-binding site, which then leads to the transcription of NF-κB target genes [18, 19]. Accordingly, Shn2 knockout (KO) mice exhibit constitutive NF-κB activation in CD4^+^ T cells [19]. Shn2 is expressed in several brain regions including the hippocampus, cortex, and cerebellum [10]. We have previously reported that Shn2 KO mice demonstrated mild, widespread brain inflammation characterized by the up-regulation of NF-κB-responsive genes and activation of astrocytes [20]. Shn2 KO mice demonstrated multiple schizophrenia-related phenotypes, including behavioral abnormalities that resemble those of schizophrenics, transcriptome/proteome changes similar to those of postmortem schizophrenia patients, decreased parvalbumin and glutamic acid decarboxylase 67 levels, increased theta power on electroencephalograms, and a thinner cortex [20]. Schizophrenic subjects are reported to have lower mRNA levels for Shn2 [21]. More recently, whole exome sequencing studies demonstrated that nine individuals with intellectual disability have distinct de novo variants in HIVEP2, and they are diagnosed with “HIVEP2 syndrome” [22, 23]. Patients with HIVEP2 syndrome exhibit intellectual disability and behavioral problems that include hyperactivity, attention-deficit disorder, aggression, anxiety, and autism spectrum disorders [22, 23]. Granule cells of the dentate gyrus (DG) failed to mature in Shn2 KO mice, a proposed endophenotype of neuropsychiatric disorders [24, 25]. Thus, the Shn2 KO mouse is an animal model for schizophrenia and intellectual disability that has good concept validity. In the brains of Shn2 KO mice, there are increases in C4b and C1q genes [20], which are thought to mediate synapse elimination during postnatal development [26, 27]. Up-regulation of C1q and C4 genes could be a potential interface between inflammation and synaptic dysfunctions. Taken together, it is of interest that the morphology of neuronal dendritic spines in Shn2 KO mice be investigated.

Serial block-face (SBF) imaging is a novel scanning electron microscopy (SEM) technique that enables much more efficient acquisition of a series of ultrastructural sectional images than previous methods such as high-voltage transmission electron microscopy (TEM) and serial-section TEM [28]. Imaging of neural tissues using SBF-SEM and three-dimensional reconstruction from serial EM images is a powerful technique for analyzing fine subcellular-scale structures [29, 30]. In the present study, we used three-dimensional reconstruction based on SBF images from SBF-SEM to analyze morphology of subcellular-scale structures in DG granule cells in Shn2 KO mice.

## Methods

### Animals

We used 10-week-old male Shn2 KO mice (*n* = 3) and their male wild-type (WT) littermates (*n* = 3). Mutant and WT mice were group housed in a room with a 12 h light/dark cycle (lights on at 7:00 a.m.), with access to food and water ad libitum. Room temperature was kept at 23 ± 2 °C. All procedures were approved by the Institutional Animal Care and Use Committee of Fujita Health University.

### Preparation of DG samples for SBF-SEM

Fixed brain samples were cut into 100-μm-thick slices with a DTK-1000 Microslicer (Dosaka EM, Kyoto, Japan), and the slices were processed largely in accordance with a combinatorial heavy metal staining protocol that has been released on the website of the National Center for Microscopy and Imaging Research (La Jolla, CA) (https://ncmir.ucsd.edu/sbem-protocol). This protocol was designed to enhance signal for backscattered electron imaging at low accelerating voltages. In brief, the tissue slices were further fixed with 2% paraformaldehyde and 2% glutaraldehyde in cacodylate buffer (0.15 M sodium cacodylate containing 2 mM CaCl_2_, pH 7.4) at 4 °C overnight. The slices were washed three times with cacodylate buffer, and then post-fixed with 2% aqueous osmium tetroxide containing 1.5% potassium ferrocyanide in cacodylate buffer for 1 h at 4 °C, filtered 1% thiocarbohydrazide solution for 20 min at room temperature, 2% osmium tetroxide solution for 30 min at room temperature, 1% aqueous uranyl acetate overnight at 4 °C, and Walton’s lead aspartate solution [31] for 30 min at 60 °C. The slices were then dehydrated with a graded series of ethanol, and then were infiltrated with durcupan resin and polymerized at 60 °C for 3 days.

### SBF-SEM

Small pieces of block including glomerulus were trimmed and mounted on aluminum SBF-SEM specimen pins (Gatan, Pleasanton, CA) using CircuitWorks Conductive Epoxy (Chemtronics, Kennesaw, GA). The entire surface of the specimen was coated with a thin layer (20 nm thickness) of gold to dissipate the electric charge caused by electron-beam irradiation during SEM imaging. In this study, we used an SBF-SEM system in which an in-chamber ultramicrotome system (3View; Gatan Inc., Pleasanton, CA) was incorporated in a SEM (MERLIN, Carl Zeiss Microscopy, Jena, Germany). SBF-SEM images were acquired as reported previously [32]. Briefly, SBF images were obtained every 50-nm depth with a backscattered electron detector at an acceleration voltage of 1.5 kV. Two different serial SEM images were recorded simultaneously. One is a large field of view image (123 μm × 246 μm) at low resolution (30 nm/pixel), including the granule cell layer used for analyses of nuclear volume and neuronal density. The other is a small field of view image (57 μm × 57 μm) at high resolution (7 nm/pixel) in the middle molecular layer (Additional file 1: Figure S1) used for detailed morphological analyses in dendrites. The contrast of the images was inversed.

### Data processing for three-dimensional reconstruction

After 2× binning of the images, the image stack was automatically aligned in a Fiji/ImageJ software package (http://fiji.sc/Fiji) as described previously [33]. Segmentation and three-dimensional reconstruction were carried out in Renovo Neural Inc. (Cleveland, OH) using Reconstruct software (https://synapseweb.clm.utexas.edu). Length and volume were generated using Reconstruct software from tracings of the dendritic shaft, dendritic spine neck, dendritic spine head, or mitochondria. Spine length or volume is the sum of length or volume of the neck and head, respectively. Diameters were calculated geometrically from length and volume assuming that each dendritic shaft, spine, spine neck, or spine head was a simple cylinder. Images and movies for figures were generated using AMIRA 5.6 Software (FEI Visualization Science Group, Burlington, MA) in Maxnet Co., Ltd. (Tokyo, Japan).

### Immunohistochemistry

Immunohistochemical analysis was performed essentially the same as previously described [20, 34]. Adult mice were deeply anesthetized and transcardially perfused with 4% paraformaldehyde in phosphate buffered saline (PBS). The brains were dissected, immersed overnight in the same fixative, and transferred to 30% sucrose in PBS for at least 3 days for cryoprotection. Brains were mounted in Tissue-Tek (Miles, Elkhart, IN), frozen, and cut into 8-μm-thick coronal sections using a microtome (CM1850; Leica Microsystems, Wetzlar, Germany). The sections were preincubated for 30 min at room temperature in 5% skim milk in PBS containing 0.05% Tween-20, and then incubated overnight at 4 °C in PBS containing the primary antibodies. We used the following primary antibodies: rabbit polyclonal antibody for GluR1 (AB1504; Millipore, Billerica, MA) and postsynaptic density 95 (PSD95) (51–6900, invitrogen, Carlsbad, CA), and mouse monoclonal antibody for synaptic vesicle 2 (SV2) (Developmental Studies Hybridoma Bank, Iowa City, IA). The antibody for GluR1 detected a single band of an expected size in Western blotting analysis using mouse brain lysates [34]. Immunoreactivity to the antigen was visualized using Alexa488- or Alexa594-conjugated secondary antibodies (Molecular Probes, Eugene, OR). Nuclear staining was performed with Hoechst 33,258 (Polyscience, Warrington, PA). We used a confocal microscope (LSM 510 META; Zeiss, Göttingen, Germany) to obtain images of the stained sections. Quantification of the immunofluorescence intensities in the DG molecular layer was performed using ZEN software (Zeiss). To quantify the expression levels of synaptic proteins, we calculated average values of immunofluorescence for WT and Shn2 KO mice by using average values of immunofluorescence for each animal. Two or three sections from each animal were processed for quantification.

### Data analysis

Statistical analysis was conducted using SAS University Edition (SAS Institute, Cary, NC). Data were analyzed using Student’s *t*-test or Wilcoxon rank sum test.

## Results

### Immature dendritic spine morphology in DG of Shn2 KO mice

**Fig. 1:**
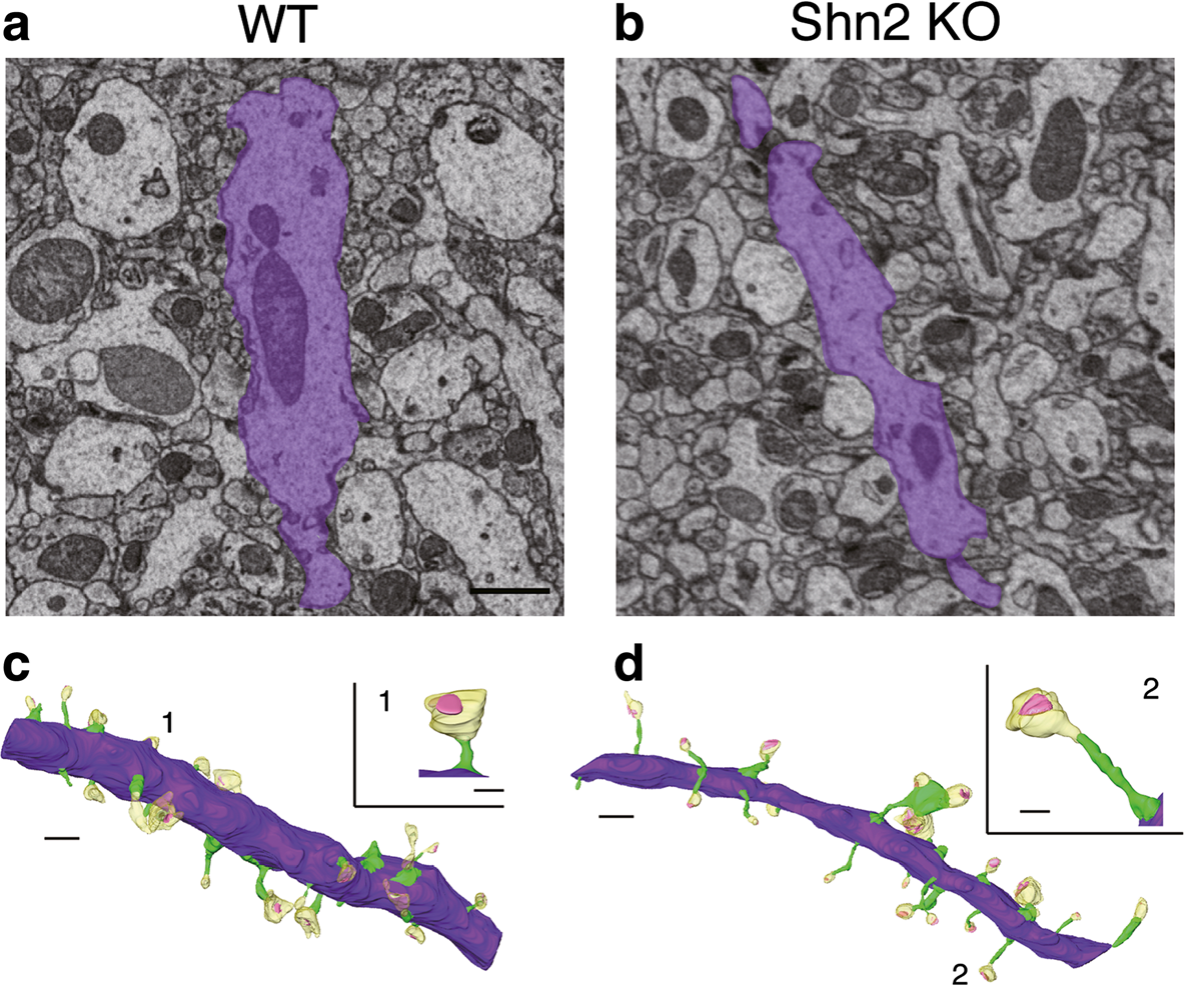
Three-dimensional reconstruction of dendrites in the DG of Shn2 KO mice. (**a**, **b**) SEM images obtained through SBF-SEM in WT (**a**) and Shn2 KO (**b**) mice. Purple shows a dendritic shaft. (**c**, **d**) Three-dimensional reconstructions of representative dendrites in WT (**a**) and Shn2 KO (**b**) mice. Scale bars: 1 μm. Representative spines are in insets. Numbers indicate the position of spines. Scale bars: 0.3 μm. Purple, green, yellow and pink illustrate the dendritic shaft, spine neck, spine head, and PSD, respectively

**Fig. 2:**
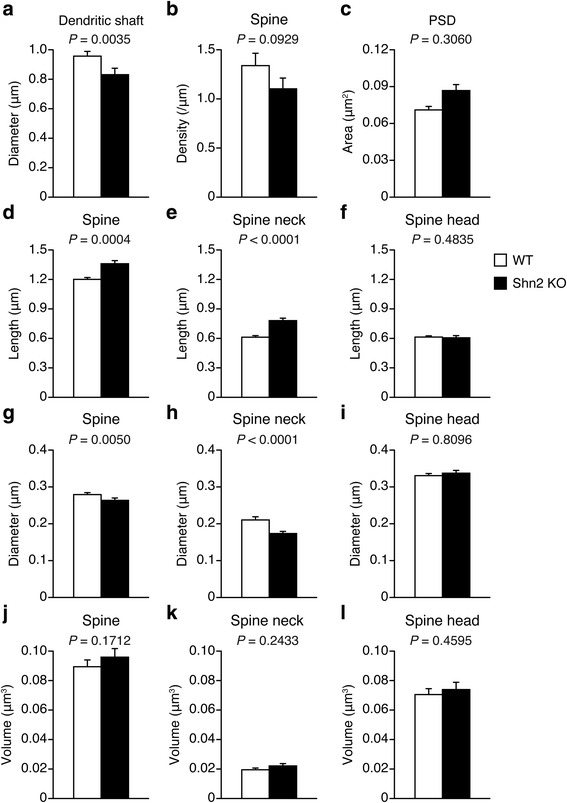
Immature dendritic spine morphology in the DG in Shn2 KO mice. (**a**, **b**) Comparison of dendrite diameter (**a**) and spine density (**b**) in WT (*n* = 24 dendrites, 8 dendrites per each of 3 mice) and Shn2 KO mice (*n* = 24 dendrites, 8 dendrites per each of 3 mice). (**c**–**l**) Mean values of PSD area (**c**), spine length (**d**), spine neck length (**e**), spine head length (**f**), spine diameter (**g**), spine neck diameter (**h**), spine head diameter (**i**), spine volume (**j**), spine neck volume (**k**), and spine head volume (**l**) in WT (*n* = 547 spines from 24 dendrites, 8 dendrites per each of 3 mice) and Shn2 KO mice (*n* = 386 spines from 24 dendrites, 8 dendrites per each of 3 mice). The *P*-values were calculated using a Wilcoxon rank sum test


We evaluated the effects of deficiency of Shn2 on the morphology of dendrites in granule cells in the middle molecular layer of the dorsal DG using serial images obtained with SBF-SEM (Additional file 1: Figure S1). Three-dimensional reconstruction of serial SEM images made it possible to visualize EM-quality ultrastructure of subcellular-scale structures. In Shn2 KO mice, thin dendrites with long and thin spines were apparent in granule cells in the middle molecular layer of the dorsal DG (Fig. 1a–d, Additional file 1: Figure S2 and Additional file 1: Figure S3, and Additional file 2: Movie S1 and Additional file 3: Movie S2). We then quantified the diameters of dendritic shaft and spine, spine density, spine length, spine volume, and postsynaptic density (PSD) area using three-dimensional reconstructed images of dendrites. Shn2 KO mice have decreased dendrite diameter compared with that of WT mice (*P* = 0.0035) (Fig. 2a). There was a non-significant tendency toward decrease in spine density per 1 μm of dendritic shaft of Shn2 KO mice (*P* = 0.0929) (Fig. 2b). PSD area was indistinguishable between Shn2 KO and WT mice (*P* = 0.3060) (Fig. 2c). The spine length of Shn2 KO mice increased over WT mice (*P* = 0.0004), which was mainly due to an increase in spine neck length in Shn2 KO mice (*P* < 0.0001) (Fig. 2d–f). Spine diameter and spine neck diameter of Shn2 KO mice were significantly lower than those of WT mice (*P* = 0.0050, and *P* < 0.0001, respectively) (Fig. 2g–i). There was no significant difference between Shn2 KO and WT mice in terms of spine volume (Fig. 2j–l).

Additional file 2: Movie S1. A movie of three-dimensional rendering of an SBF-SEM dataset from DG in WT mouse. Purple, green, yellow, and pink illustrate the dendritic shaft, spine neck, spine head, and PSD, respectively. (MOV 8.69 mb)



Additional file 3: Movie S2. A movie of three-dimensional rendering of an SBF-SEM dataset from DG in Shn2 KO mouse. Purple, green, yellow, and pink illustrate dendritic shaft, spine neck, spine head, and PSD, respectively. (MOV 9315 kb)


### Decreased expression levels of synaptic proteins in the DG of Shn2 KO mice

**Fig. 3:**
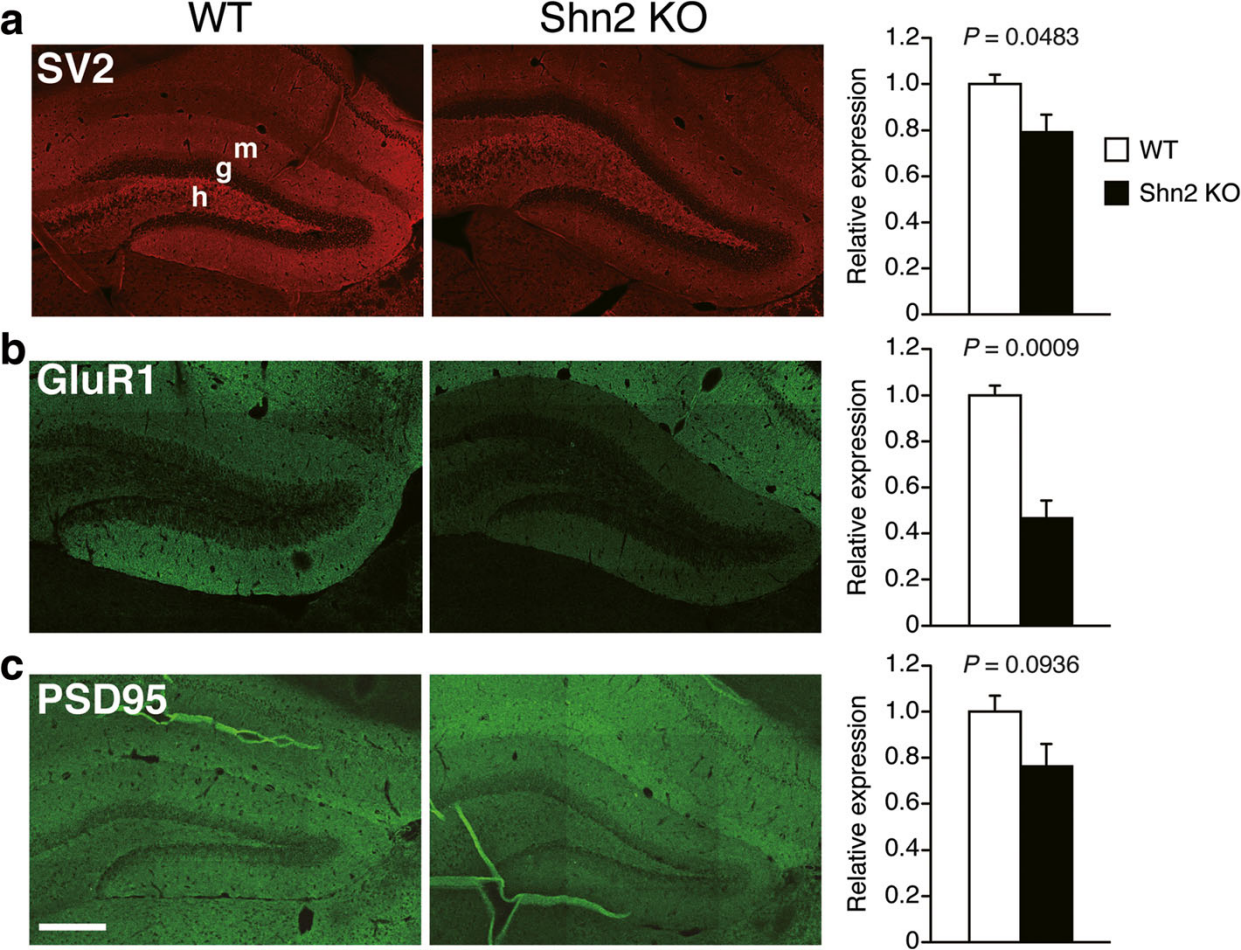
Decreased expression of GluR1 and a nominally significant decrease in SV2 expression in the middle molecular layer of the DG of Shn2 KO mice. (**a**–**c**) Representative images of SV2 (**a**), GluR1 (**b**), and PSD95 (**c**) staining in the DG of WT and Shn2 KO mice. Bar graphs represent fluorescence intensity normalized to that in the middle molecular layer in the DG of WT mice, and are presented as the mean ± SEM. For WT, *n* = 4 mice; for Shn2 KO, *n* = 4 mice. The *P*-values were calculated using Student’s *t*-test. Scale bar, 300 μm; g, granule cell layer; h, hilus; m, molecular layer

Since Shn2 KO mice showed abnormalities in spine morphology, we then conducted immunofluorescence analyses using laser scanning confocal microscopy to evaluate expression levels of synaptic proteins, such as SV2, GluR1, and PSD95, in hippocampal regions in Shn2 KO mice (Fig. 3 and Additional file 1: Figure S4). In the middle molecular layer of the DG, in which SBF-SEM analyses were conducted, Shn2 KO mice showed decreased expression levels of SV2 (*P* = 0.0483) and GluR1 (*P* = 0.0009) compared with those in WT mice (Fig. 3a and b). PSD95 expression in the middle molecular layer had a non-significant tendency to decrease in Shn2 KO mice (*P* = 0.0936; Fig. 3c). In the inner molecular layer of Shn2 KO mice, expressions of SV2, GluR1, and PSD95 were significantly decreased (Additional file 1: Figure S4a–c; *P* = 0.0149, 0.0015, and 0.0481, respectively). In the outer molecular layer of Shn2 KO mice, GluR1 expression was significantly decreased (*P* = 0.0007; Additional file 1: Figure S4e), while SV2 and PSD95 were not significantly different between Shn2 KO and WT mice (Additional file 1: Figure S4d and f; *P* = 0.0573 and 0.1024, respectively). In the CA1 radiatum layer, there were no significant differences between Shn2 KO and WT mice in the expression levels of SV2, GluR1, and PSD95 (Additional file 1: Figure S4g–i; *P* = 0.7262, 0.1435, and 0.8364, respectively). To avoid false-positive results caused by the multiple statistical tests performed, Bonferroni correction was applied to these results (the adjusted *P*-value at the 0.05 significance level for 12 indices was 0.004167). After the correction, the results remained significant for expression levels of GluR1 in the outer, middle, and inner molecular layers of the DG in Shn2 KO mice, while the other expression differences did not survive (Fig. 3 and Additional file 1: Figure S4). These results demonstrated that Shn2 KO mice showed significantly decreased expression levels of GluR1 in the whole molecular layer, whereas there were nominally significant decreases in SV2 and PSD95 in specific regions of the molecular layer in the DG of Shn2 KO mice.

### Abnormal mitochondrial morphology in Shn2 KO mice

**Fig. 4:**
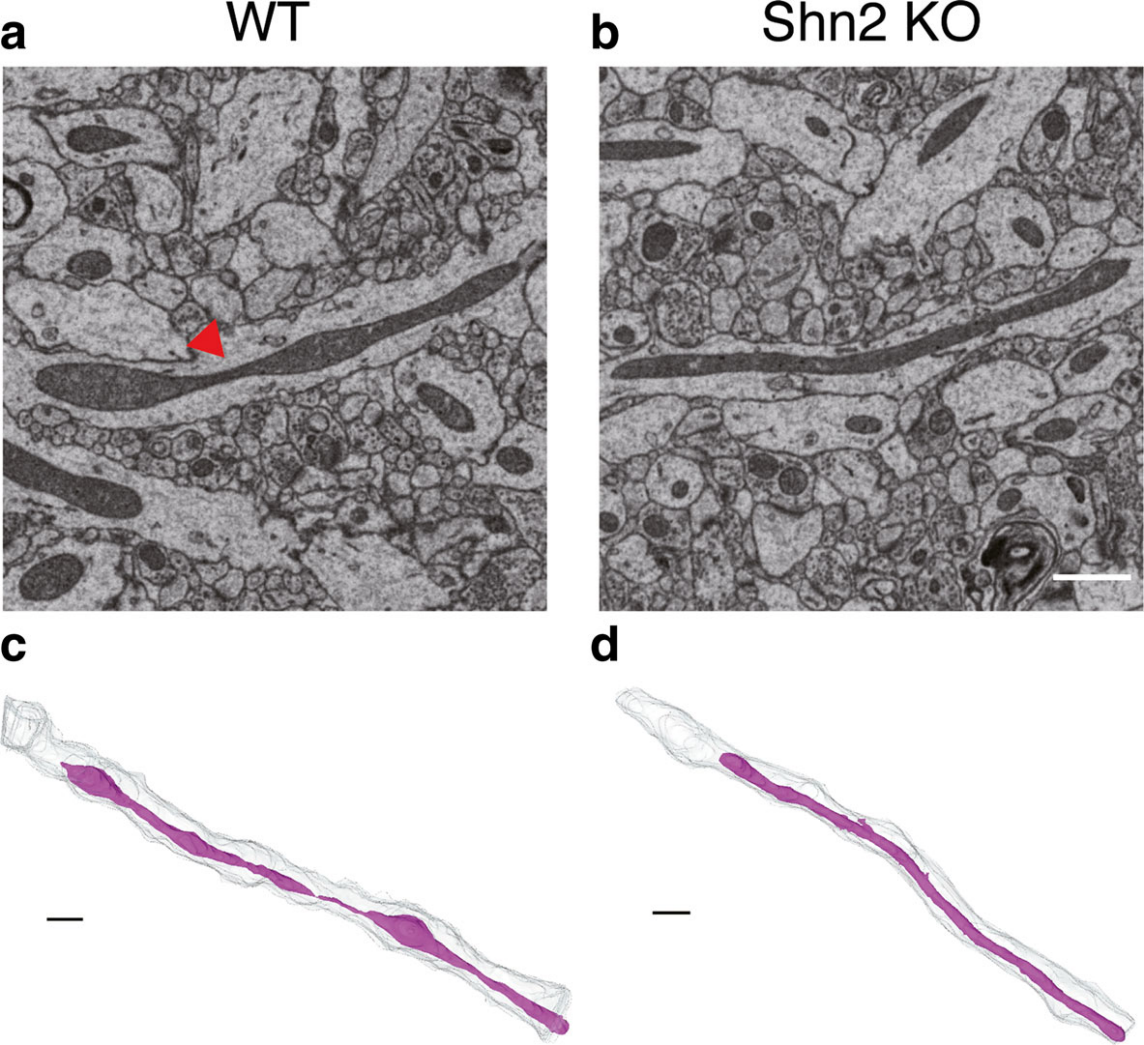
Abnormal mitochondrial morphology in Shn2 KO mice. (**a**, **b**) SEM images of mitochondria in WT (**a**) and Shn2 KO (**b**) mice. Red triangle indicates constriction in a mitochondrion. Scale bars, 1 μm. (**c**, **d**) Three-dimensional reconstruction of representative constricted mitochondrion in WT (**c**) and elongated mitochondrion in Shn2 KO (**d**) mice. Magenta shows a mitochondrion. Grey indicates dendritic shafts

SEM images show that filamentous mitochondria are found in dendritic shaft (Fig. 4a), which is consistent with previous reports [35, 36]. SEM images and three-dimensional reconstruction of mitochondria suggested that ratios of constricted mitochondria to elongated ones were different between Shn2 KO and WT mice (Fig. 4). We counted the number of constricted and elongated mitochondria whose volumes were greater than 0.1 μm^3^, since mitochondria whose volumes were less than 0.1 μm^3^ demonstrated a spherical shape. In the mutants, the number of constricted mitochondria was decreased compared with those of WT (χ^2^ = 6.9873, *P* = 0.0082) (Table 1). Volumetric comparisons demonstrated that there were no significant differences between Shn2 KO and WT mice in mitochondria volume, mitochondria length, or mitochondria number per 1 μm of dendritic shaft (Additional file 1: Figure S5a–c; *P* = 0.7356, 0.1083, and 0.1703, respectively).

**Table 1:** Abnormal mitochondrial morphology in Shn2 KO mice

	Constricted mitochondria	Elongated mitochondria	Total
WT	27	33	60
Shn2 KO	8	33	41
Total	35	66	101

Cross tabulation of the number of constricted and elongated mitochondria in WT (*n* = 60 mitochondria from 24 dendrites, 8 dendrites per each of 3 mice) and Shn2 KO mice (*n* = 41 mitochondria from 24 dendrites, 8 dendrites per each of 3 mice). This cross tabulation yielded a significant chi-square value (χ^2^ = 6.9873, *P* = 0.0082)

### Decreased nuclear volume and increased neuronal density in Shn2 KO mice

**Fig. 5:**
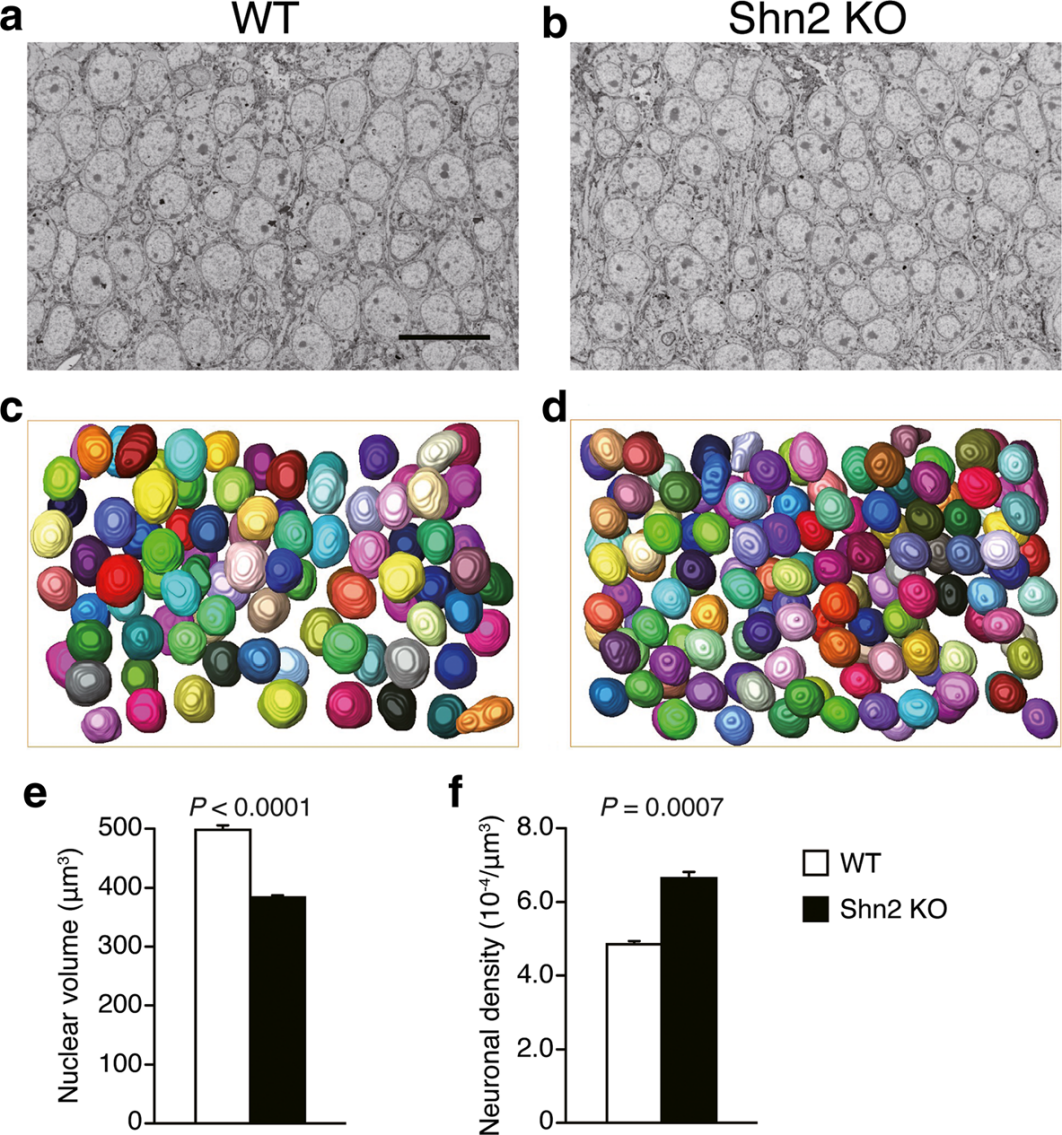
Decreased nuclear volume and increased neuronal density in Shn2 KO mice. (**a**, **b**) SEM images of cell bodies of granule cells in WT (**a**) and Shn2 KO (**b**) mice. Scale bars, 20 μm (**c**, **d**) Three-dimensional reconstruction of nuclear in granule cells of WT (**c**) and Shn2 KO (**d**) mice. (**e**) Comparison of neuronal density in WT (*n* = 3) and Shn2 KO (*n* = 3) mice. (**f**) Comparison of nuclear volume in WT (*n* = 120 from 3 mice) and Shn2 KO mice (*n* = 120 from 3 mice). The *P*-values were calculated using Student’s *t*-test (**e**) and Wilcoxon rank sum test (**f**)

SEM images suggest decreased cell body size in the granule cell layer of DG in Shn2 KO mice (Fig. 5a and b). To assess cell body size, nuclei of granule cells were reconstructed in three dimensions (Fig. 5c and d). There was a significant decrease in nuclear volume in the mutants over WT mice (*P* < 0.0001) (Fig. 5e). Neuronal density calculated from the number of nuclei was significantly increased in Shn2 KO mice compared with that in WT mice (*P* = 0.0007) (Fig. 5f), which is consistent with a previous report that cell-packing density was higher in the DG of Shn2 KO mice [20].

## Discussion and conclusions

**Fig. 6:**
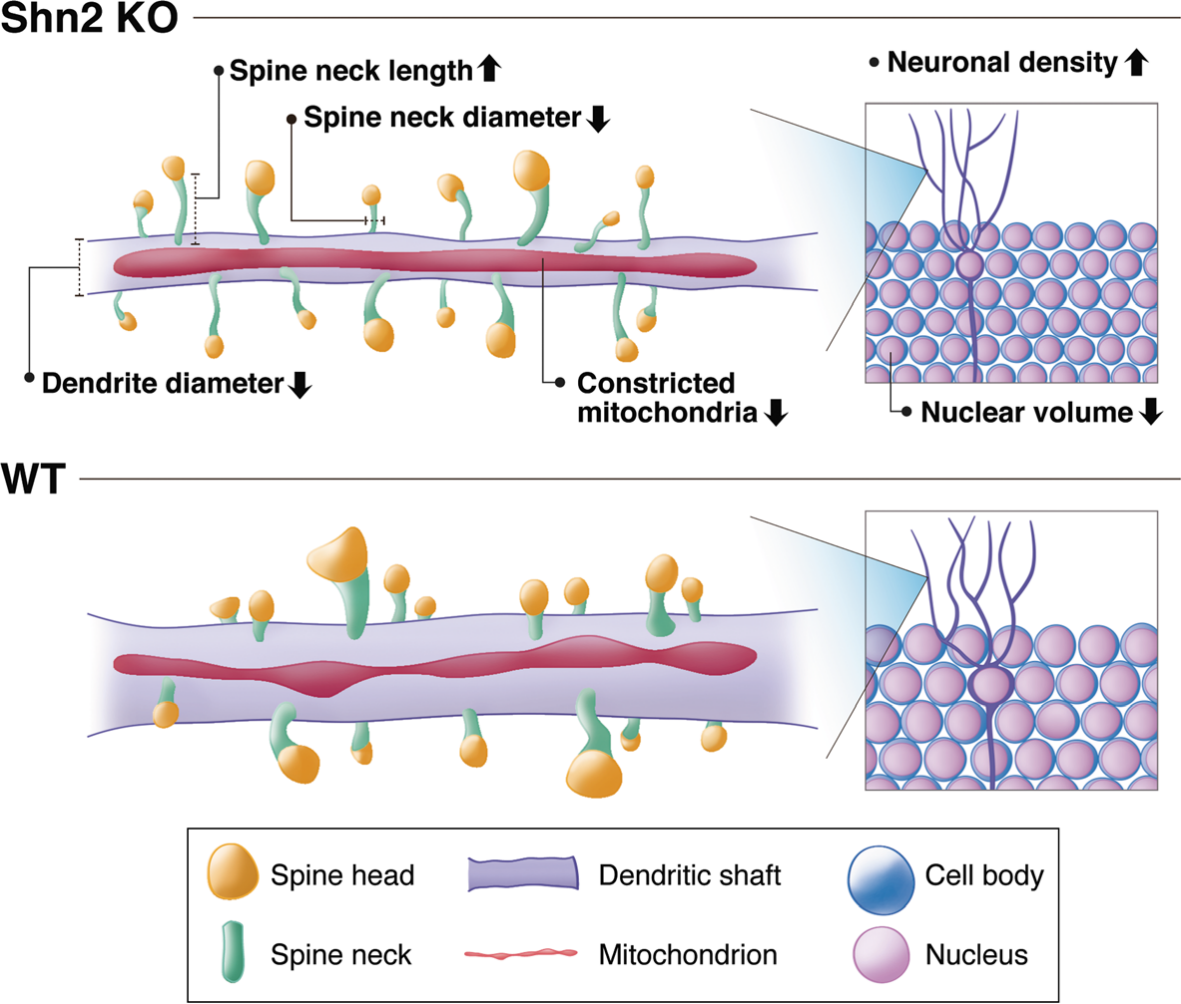
Schematic of morphological properties of subcellular-scale structures in DG of Shn2 KO mice

Imaging of neural tissues using SBF-SEM and three-dimensional reconstruction from serial EM images is a powerful technique for analyzing subcellular-scale structures. Using this technique, we identified novel morphological phenotypes in Shn2 KO DG (a schematic is provided in Fig. 6). Shn2 KO mice have long and thin spines resembling immature spine-like structures called filopodia, which may later evolve into dendritic spines [37]. Shn2 KO mice showed a significant reduction in GluR1 expression, and a nominally significant decrease in SV2 expression. PSD95 expression had a non-significant tendency to decrease in Shn2 KO mice, which is consistent with the finding of a non-significant tendency for decreased spine density in Shn2 KO mice compared with that in WT mice. The mutants exhibited decreased numbers of constricted mitochondria, suggesting that a balance between mitochondrial fusion and fission is compromised in Shn2 KO mice. Additionally, there were significant decreases in dendrite diameter and nuclear volume in the mutants. Neuronal density in Shn2 KO mice was increased compared with that in WT mice.

An “immature DG (iDG)” was first discovered in α-CaMKII^+/−^ mice, which display abnormal behaviors related to schizophrenia, bipolar disorder, and other psychiatric disorders [25, 38]. Previously, we have reported that Shn2 KO mice also possessed an iDG phenotype in terms of gene expression pattern and electrophysiological properties [20]. The present study shows morphological immaturity of the dendritic spine in the DG of Shn2 KO mice, which is consistent with the idea that Shn2 KO mice have iDG phenotypes. Shn2 KO mice showed decreased expression levels of GluR1 in the entire molecular layer of the DG. GluR1 is expressed primarily in mature granule cells, and can be a potential marker for mature granule cells [34]. It has been reported that GluR1 expression is reduced in the DG of α-CaMKII^+/−^ mice [34]. GluR1 reduction may be one of the shared changes among a subgroup of iDG mice. In the inner molecular layers of the DG in Shn2 KO mice, there were statistically significant reductions in GluR1 expression and nominally significant decreases in SV2 and PSD95, suggesting an altered number of synapses. Further SBF-SEM analysis is necessary to clarify the morphological changes in the inner molecular layer of the DG in Shn2 KO mice. Treatment with chronic fluoxetine—a selective serotonin reuptake inhibitor—reverses the neuronal maturation, resulting in the iDG phenotype [39]. Kitahara et al. carried out a morphological analysis of synapses in the molecular layer of the DG after chronic fluoxetine treatment using focused ion beam-SEM [40]. They showed that spine density is indistinguishable between chronic fluoxetine treatment and placebo mice, which is consistent with the result in Shn2 KO mice. While there was no significant difference between Shn2 KO and WT mice in spine volume and PSD area, chronic fluoxetine treatment increased spine and PSD volume compared with placebo mice. Further studies are required to investigate the effects of shared and unshared morphological changes among iDG mice in dendritic spines on neuronal function.

We previously showed significant increases in expression level of genes involved in immune responses, such as complement genes in Shn2 KO mice [20]. Notably, C1qa, C1qb, C1qc, and C4 were up-regulated in the brains of Shn2 KO mice. Complement proteins are widely expressed in neurons and glia in postnatal brain, and they play an important role in a process critical for establishing precise synaptic circuits [27, 41]. Sekar et al. reported that association of schizophrenia with the MHC locus arises in part from many structurally diverse alleles of C4 genes, and expression of those genes is increased in post-mortem brains of schizophrenic patients [26]. They proposed that excessive complement activity might explain the reduced number of synapses in brains of individuals with schizophrenia. In addition, our previous study showed decreased gene expression of kalirin in the DG of Shn2 KO mice [20]. Kalirin is a schizophrenia-associated gene, and loss of kalirin correlates with decreased spine density in prefrontal cortex neurons [3]. However, spine density was statistically indistinguishable between Shn2 KO and WT mice. It is noteworthy that the level of brain-derived neurotrophic factor (BDNF)—a neurotrophin protein—is increased in the DG of Shn2 KO mice [42]. BDNF stimulation is reported to increase the density of dendritic filopodia and spines [43]. It is possible that Shn2 KO mice have an enhanced turnover of spines due to an increase in expression of BDNF. Interestingly, patients with fragile X syndrome, intellectual disability-related diseases, exhibited an immature dendritic spine morphology and an increased dendritic spine density, suggesting enhanced spine turnover [44]. It has been reported that Fmr1 KO mice, a fragile X syndrome mouse model, displayed enhanced spine turnover [45]. Dendritic spine morphology in Shn2 KO DG neurons recapitulates that seen in fragile X syndrome. Thus, the Shn2 KO mouse may be a good model for investigating spine morphology in intellectual disability.

It is thought that mitochondrial dysfunction may underlie the pathophysiology of schizophrenia, bipolar disorder, and intellectual disability [2, 9]. Shn2 KO mice exhibited a decreased number of constricted mitochondria over WT mice, suggesting that a balance between mitochondrial fusion and fission is compromised in the mutants. Interestingly, Zhao and Li showed that lack of dysbindin, a schizophrenia susceptibility gene [46], increased mitochondrial fission through dynamin-related protein 1 [47]. Mitochondrial fission and fusion play critical roles in maintaining functional mitochondria when cells experience metabolic or environmental stresses [48]. In the DG of Shn2 KO mice, genechip analysis demonstrated altered gene expression related to energy metabolism such as aldo-keto reductase genes *Akr1c18*, ATP-related genes (*Atp2a*, *Atp2b*, *Atp6v,* and *Atp11c*), the cytochrome-related gene *Cyb561*, and the phosphoglycerate-related gene *Phgdh* [20]. Changes in protein amounts were also found in energy metabolism-related molecules, including aldo-keto reductase genes *Akr1b3*, ATP-related genes (*Atp5a*, *Atp5d*, *Atp5f*, *Atp5k*, and *Atp6v*), cytochrome-related genes (*Uqcrfs1*, *Ubcrc2*, *Cox5b*, *Uqcrb*, and *Uchl1*), the NADH dehydrogenase gene *Ndufa10*, and the phosphoglycerate-related gene *Pgk1* in the DG of Shn2 KO mice [20]. These gene/protein expression changes may be related to abnormal mitochondrial morphology in Shn2 KO mice. Further studies are needed to investigate the relationships between morphological abnormality of mitochondria and energy metabolism-related gene/protein expression changes in Shn2 KO mice. Recently, we reported that significantly lower pH and higher lactate levels were observed in brains of mouse models for psychiatric disorders, including Shn2 KO mice, as well as a significant negative correlation between pH and lactate levels [49]. We proposed that elevated glycolysis underlies increases in lactate levels, which is similar to the Warburg effect. Taken together, metabolism in Shn2 KO mice may be compromised due to abnormal mitochondrial morphology and metabolism-related gene/protein expression changes. It is possible that morphological abnormality in mitochondria is one of the shared endophenotypes in subgroups of psychiatric disorders.

Shn2 KO mice showed decreased nuclear volume in DG. In Shn2 KO mice, adult neurogenesis is enhanced in DG (Hagihara et al.*,* unpublished observation). The present study confirmed that neuronal density in Shn2 KO mice was increased compared with that in WT mice, which is consistent with a previous report [20]. It is possible that decreased nuclear volume in the DG of Shn2 KO mice may be due to increased neuronal density caused by enhanced neurogenesis. Our results are consistent with previous reports that show reduced hippocampal neuronal size in postmortem brains of schizophrenia patients [50].

Taken together, morphological changes in Shn2 KO DG neurons recapitulate some aspects of morphological changes in psychiatric disorders such as schizophrenia and intellectual disability. Thus, Shn2 KO mice serve as a unique tool for investigating morphological abnormalities of subcellular-scale structures in those disorders and their related diseases.

## Additional files

Additional file 1: **Figure S1.** Analysis area of the middle molecular layer of the dorsal DG for SBF-SEM imaging. (a) A schematic of the sampling area (red square) at a distance of approximately 100 μm from the upper blade of the granule cell layer. OML, outer molecular layer; MML, middle molecular layer; IML, inner molecular layer; GCL, granule cell layer. (b) Boxed regions indicate the tissue area sampled used for detailed morphological analyses in the dendrites in three WT mice and three Shn2 KO mice. Scale bar: 100 μm. **Figure S2.** Three-dimensional reconstruction of all dendrites for analysis in WT mice. Dendrite segments (white transparent) are illustrated with mitochondria (blue) and spines (head, orange; neck, green; PSD, magenta). Eight dendrites per each of three WT mice. **Figure S3.** Three-dimensional reconstruction of all dendrites for analysis in Shn2 KO mice. Dendrite segments (white transparent) are illustrated with mitochondria (blue) and spines (head, orange; neck, green; PSD, magenta). Eight dendrites per each of three Shn2 KO mice. **Figure S4.** Decreased expression levels of synaptic proteins in the DG of Shn2 KO mice (a–i) Bar graphs of SV2, GluR1, and PSD95 in the inner (a–c) and outer (d–f) molecular layers of the DG, and CA1 radiatum layer (d–f) represent fluorescence intensity normalized to that of WT mice, and are presented as the mean ± SEM. IML, inner molecular layer; OML, outer molecular layer; Rad, radiatum layer. For WT, n = 4 mice; for Shn2 KO, n = 4 mice. The *P*-values were calculated using Student’s *t*-test. **Figure S5.** Volumetric comparisons of mitochondria in WT and Shn2 KO mice. Comparison of mitochondria volume (a), mitochondria length (b), and mitochondria number per 1 μm of dendrite (c) in WT (n = 96 mitochondria from 24 dendrites, 8 dendrites per each of 3 mice) and Shn2 KO mice (n = 57 mitochondria from 24 dendrites, 8 dendrites per each of 3 mice). The *P*-values were calculated using Wilcoxon rank sum test. (DOC 8 mb)

## Abbreviations

BDNF: Brain-derived neurotrophic factor
DG: Dentate gyrus
HIVEP2: Human immunodeficiency virus type I enhancer binding protein 2
iDG: Immature dentate gyrus
KO: Knockout
MHC: Major histocompatibility complex
NF-κB: Nuclear factor-κB
PBS: Phosphate buffered saline
PSD: Postsynaptic density 26
PSD95: Postsynaptic density 95
SBF-SEM: serial block-face-scanning electron microscopy
Shn2: Schnurri-2
SV2: Synaptic vesicle 2
TEM: Transmission electron microscopy
