# Supplementary material for: Immature morphological properties in subcellular-scale structures in the dentate gyrus of Schnurri-2 knockout mice: a model for schizophrenia and intellectual disability
Source: Mol Brain. 2017 Dec 12;10:60. doi: 10.1186/s13041-017-0339-2 (PMC5727961; doi:10.1186/s13041-017-0339-2)

***Supplementary information***

**Immature morphological properties in subcellular-scale structures in the dentate gyrus of Schnurri-2 knockout mice: a model for schizophrenia and intellectual disability**

**Akito Nakao, PhD^1^; Naoyuki Miyazaki, PhD^2^; Koji Ohira, PhD^3^; Hideo Hagihara, PhD^1^; Tsuyoshi Takagi, PhD^4,5^;** **Nobuteru Usuda, MD, PhD^6^; Shunsuke Ishii, PhD^5^;** **Kazuyoshi Murata, PhD^2^ and Tsuyoshi Miyakawa, PhD^1^***

^1^Division of Systems Medical Science, Institute for Comprehensive Medical Science, Fujita Health University, Toyoake, Japan

^2^National Institute for Physiological Sciences, National Institutes of Natural Sciences, Okazaki, Japan

^3^Department of Food Science and Nutrition, Mukogawa Women’s University, Nishinomiya, Japan

^4^Institute for Developmental Research, Aichi Human Service Center, Kasugai, Japan

^5^RIKEN Tsukuba Institute, Tsukuba, Japan

^6^Department of Anatomy II, Fujita Health University School of Medicine, Toyoake, Japan

*** Correspondence:**

Tsuyoshi Miyakawa

Division of Systems Medical Science, Institute for Comprehensive Medical Science, Fujita Health University, 1-98 Dengakugakubo, Kutsukake-cho, Toyoake, Aichi 470-1192, Japan

E-mail: miyakawa@fujita-hu.ac.jp

**Supplementary Figure 1 Analysis area of the middle molecular layer of the dorsal DG for SBF-SEM imaging.** (a) A schematic of the sampling area (red square) at a distance of approximately 100 μm from the upper blade of the granule cell layer. OML, outer molecular layer; MML, middle molecular layer; IML, inner molecular layer; GCL, granule cell layer. (b) Boxed regions indicate the tissue area sampled used for detailed morphological analyses in the dendrites in three WT mice and three Shn2 KO mice. Scale bar: 100 μm.

**Supplementary Figure 2 Three-dimensional reconstruction of all dendrites for analysis in WT mice.** Dendrite segments (white transparent) are illustrated with mitochondria (blue) and spines (head, orange; neck, green; PSD, magenta). Eight dendrites per each of three WT mice.

**Supplementary Figure 3 Three-dimensional reconstruction of all dendrites for analysis in Shn2 KO mice.** Dendrite segments (white transparent) are illustrated with mitochondria (blue) and spines (head, orange; neck, green; PSD, magenta). Eight dendrites per each of three Shn2 KO mice.

**Supplementary Figure 4 Decreased expression levels of synaptic proteins in the DG of Shn2 KO mice**

(a–i) Bar graphs of SV2, GluR1, and PSD95 in the inner (a–c) and outer (d–f) molecular layers of the DG, and CA1 radiatum layer (d–f) represent fluorescence intensity normalized to that of WT mice, and are presented as the mean ± SEM. IML, inner molecular layer; OML, outer molecular layer; Rad, radiatum layer. For WT, n = 4 mice; for Shn2 KO, n = 4 mice. The *P*-values were calculated using Student’s *t*-test.

**Supplementary Figure 5 Volumetric comparisons of mitochondria in WT and Shn2 KO mice.** Comparison of mitochondria volume (a), mitochondria length (b), and mitochondria number per 1 μm of dendrite (c) in WT (n = 96 mitochondria from 24 dendrites, 8 dendrites per each of 3 mice) and Shn2 KO mice (n = 57 mitochondria from 24 dendrites, 8 dendrites per each of 3 mice). The *P*-values were calculated using Wilcoxon rank sum test.

**Supplementary Movie 1 A movie of three-dimensional rendering of an SBF-SEM dataset from DG in WT mouse.** Purple, green, yellow, and pink illustrate the dendritic shaft, spine neck, spine head, and PSD, respectively.

**Supplementary Movie 2 A movie of three-dimensional rendering of an SBF-SEM dataset from DG in Shn2 KO mouse.** Purple, green, yellow, and pink illustrate dendritic shaft, spine neck, spine head, and PSD, respectively.


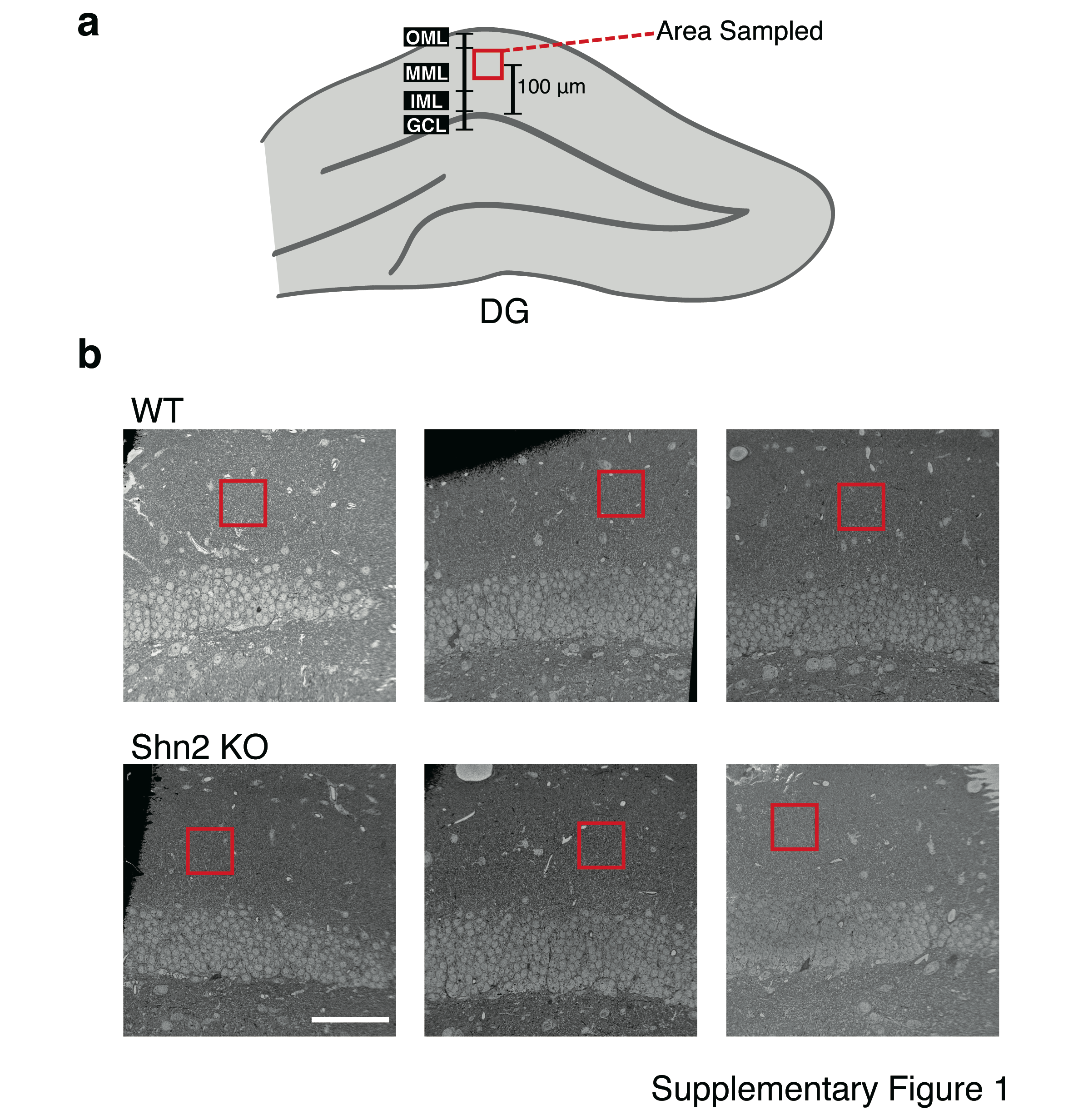


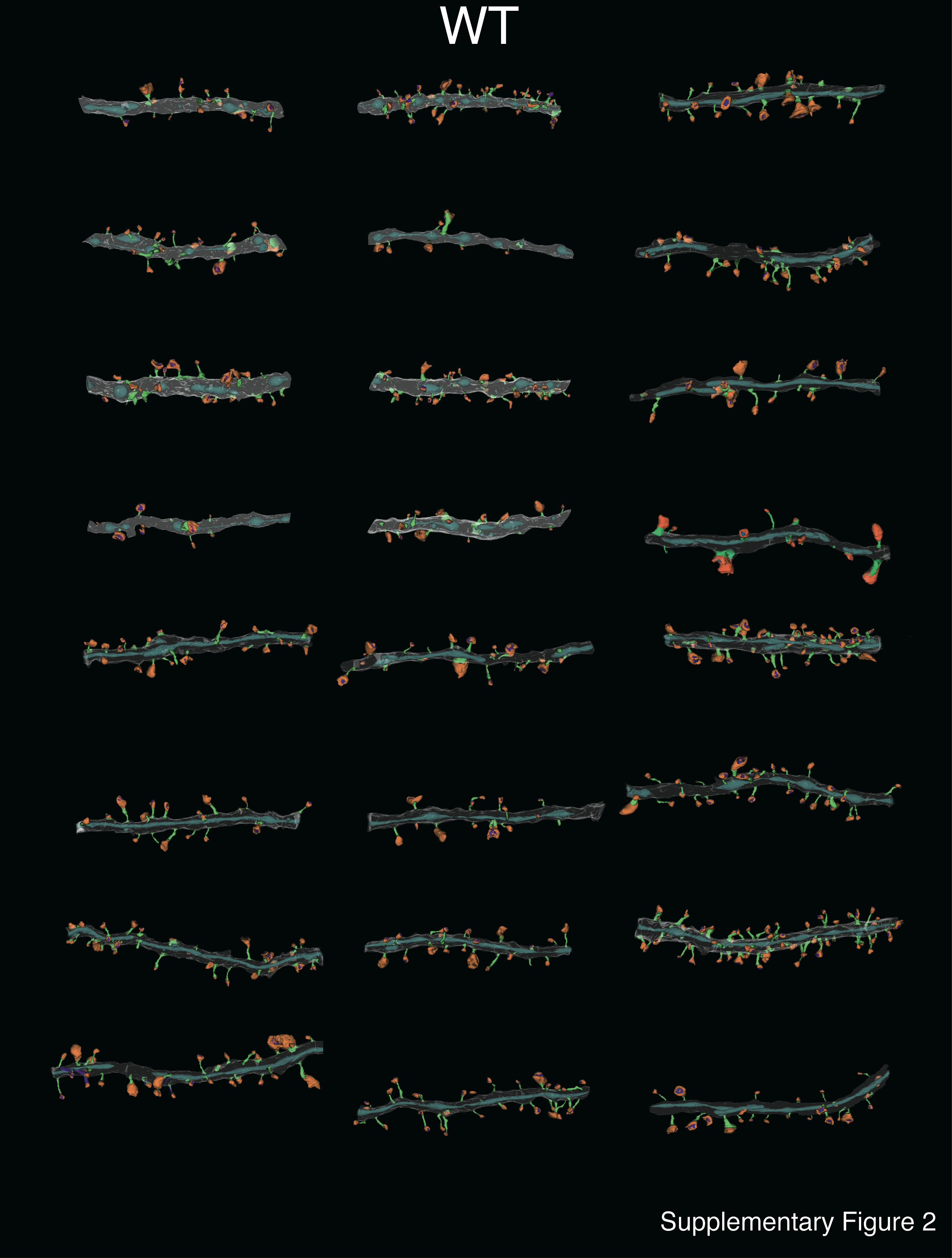


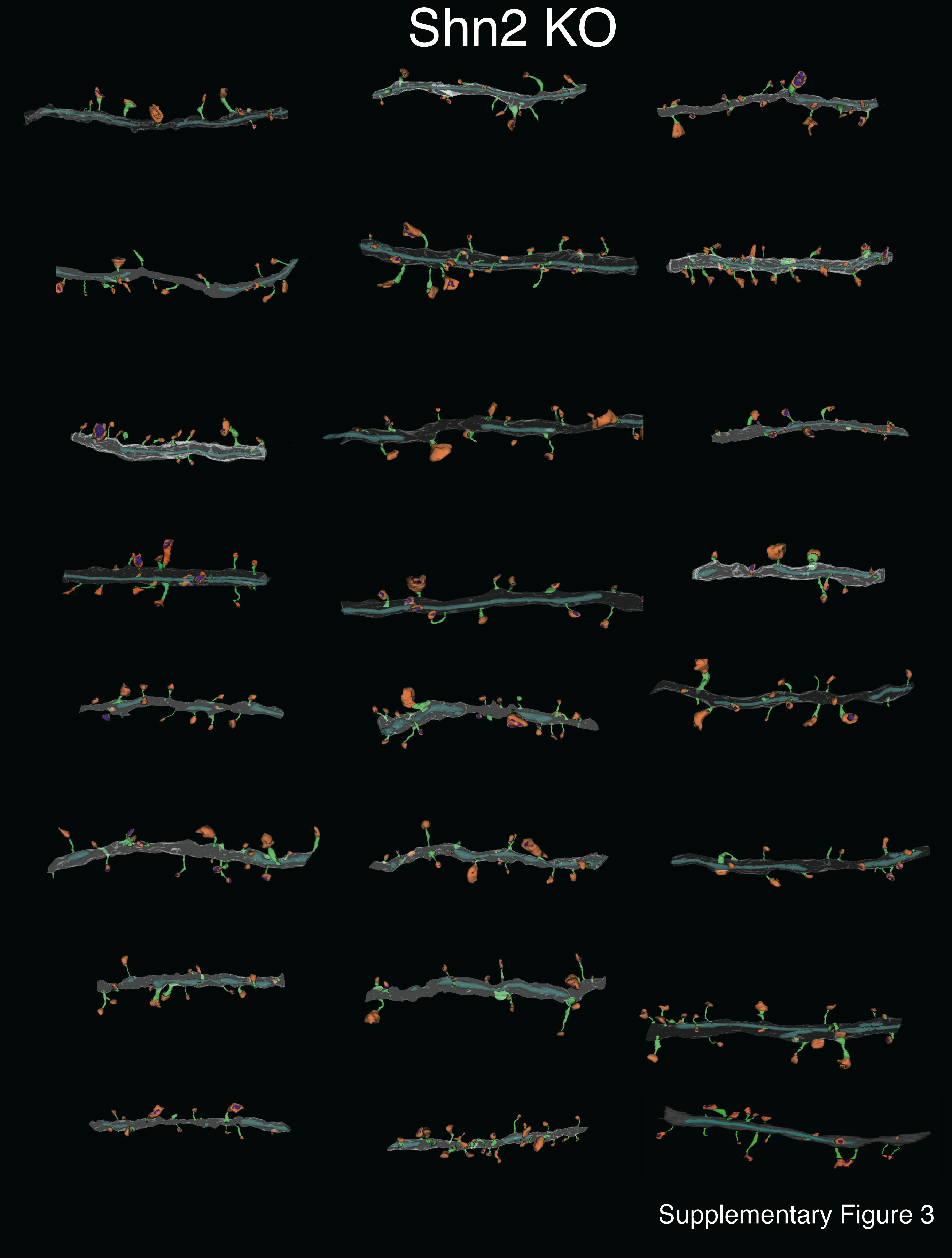


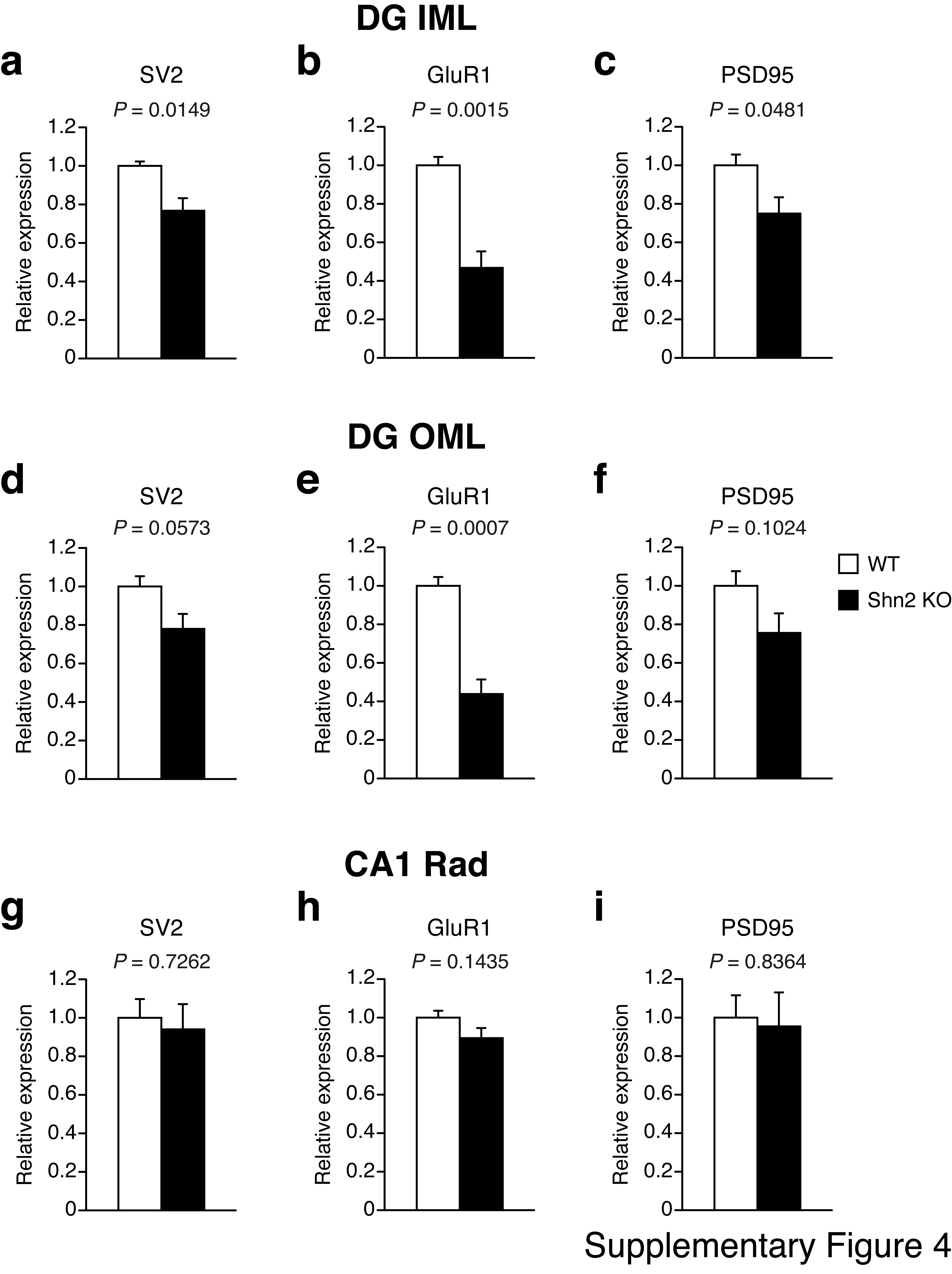


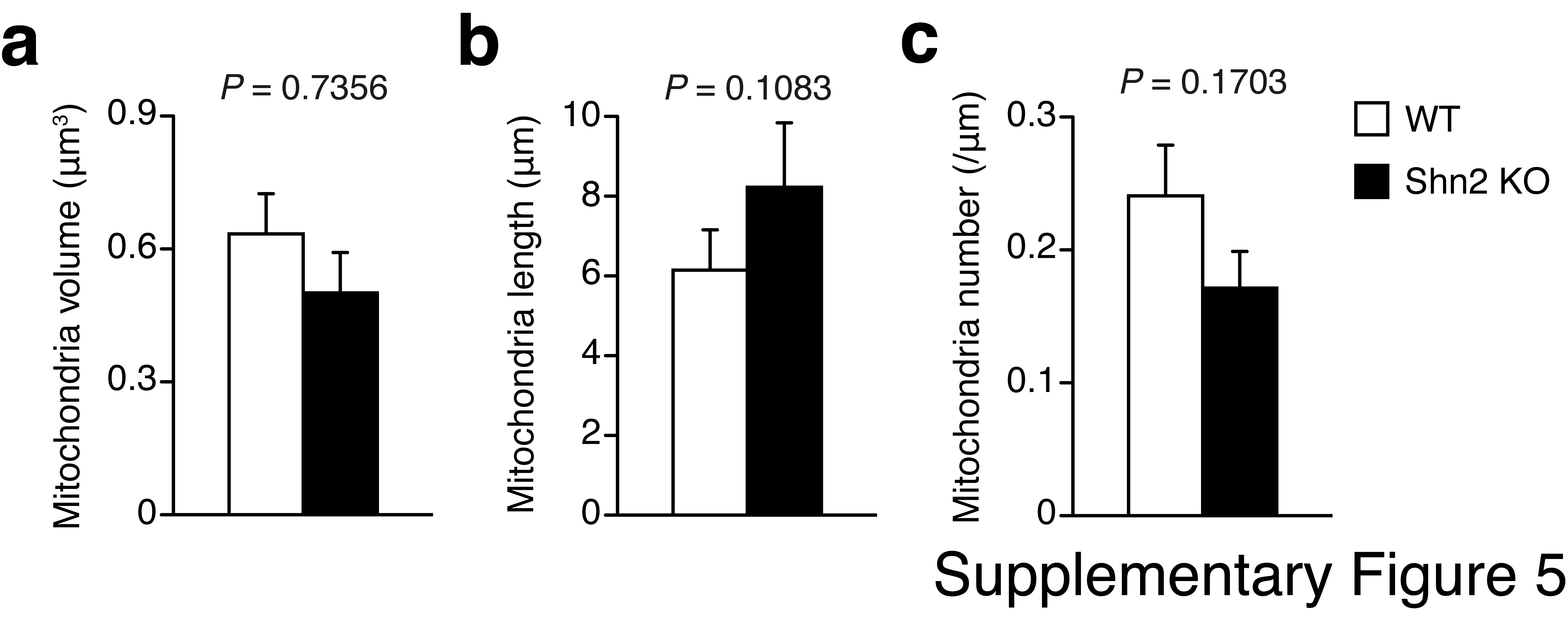

Supplement: Supplementary file 3 — Figure S1. Analysis area of the middle molecular layer of the dorsal DG for SBF-SEM imaging. (a) A schematic of the sampling area (red square) at a distance of approximately 100 μm from the upper blade of the granule cell layer. OML, outer molecular layer; MML, middle molecular layer; IML, inner molecular layer; GCL, granule cell layer. (b) Boxed regions indicate the tissue area sampled used for detailed morphological analyses in the dendrites in three WT mice and three Shn2 KO mice. Scale bar: 100 μm. Figure S2. Three-dimensional reconstruction of all dendrites for analysis in WT mice. Dendrite segments (white transparent) are illustrated with mitochondria (blue) and spines (head, orange; neck, green; PSD, magenta). Eight dendrites per each of three WT mice. Figure S3. Three-dimensional reconstruction of all dendrites for analysis in Shn2 KO mice. Dendrite segments (white transparent) are illustrated with mitochondria (blue) and spines (head, orange; neck, green; PSD, magenta). Eight dendrites per each of three Shn2 KO mice. Figure S4. Decreased expression levels of synaptic proteins in the DG of Shn2 KO mice (a–i) Bar graphs of SV2, GluR1, and PSD95 in the inner (a–c) and outer (d–f) molecular layers of the DG, and CA1 radiatum layer (d–f) represent fluorescence intensity normalized to that of WT mice, and are presented as the mean ± SEM. IML, inner molecular layer; OML, outer molecular layer; Rad, radiatum layer. For WT, n = 4 mice; for Shn2 KO, n = 4 mice. The P-values were calculated using Student’s t-test. Figure S5. Volumetric comparisons of mitochondria in WT and Shn2 KO mice. Comparison of mitochondria volume (a), mitochondria length (b), and mitochondria number per 1 μm of dendrite (c) in WT (n = 96 mitochondria from 24 dendrites, 8 dendrites per each of 3 mice) and Shn2 KO mice (n = 57 mitochondria from 24 dendrites, 8 dendrites per each of 3 mice). The P-values were calculated using Wilcoxon rank sum test. (DOC 8 mb) [file 13041_2017_339_MOESM1_ESM.doc]
